# General and Intensive Care Outcomes for Hospitalized Patients With Solid Organ Transplants With COVID-19

**DOI:** 10.1177/0885066620965163

**Published:** 2020-10-09

**Authors:** Fiore Mastroianni, Daniel E. Leisman, Grace Fisler, Mangala Narasimhan

**Affiliations:** 1 Division of Pulmonary, Critical Care, and Sleep Medicine, Department of Medicine, 232890 Donald and Barbara Zucker School of Medicine at Hofstra/Northwell, Northwell Health, New York, NY, USA; 2 5925 Icahn School of Medicine at Mount Sinai, New York, NY, USA; 3 Department of Medicine, Massachusetts General Hospital, Harvard Medical School, Boston, MA, USA; 4 Division of Pediatric Critical Care Medicine, Department of Pediatrics, 232890 Donald and Barbara Zucker School of Medicine at Hofstra/Northwell, Northwell Health, New York, NY, USA

**Keywords:** critical care, endotracheal intubation, infections, respiratory failure

## Abstract

**Purpose:**

COVID-19 has been associated with a dysregulated inflammatory response. Patients who have received solid-organ transplants are more susceptible to infections in general due to the use of immunosuppressants. We investigated factors associated with mechanical ventilation and outcomes in solid-organ transplant recipients with COVID-19.

**Materials and Methods:**

We conducted a retrospective cohort study of all solid-organ transplant recipients admitted with a diagnosis of COVID-19 in our 23-hospital health system over a 1-month period. Descriptive statistics were used to describe hospital course and laboratory results and bivariate comparisons were performed on variables to determine differences.

**Results:**

Twenty-two patients with solid-organ transplants and COVID-19 were identified. Eight patients were admitted to the ICU, of which 7 were intubated. Admission values of CRP (p = 0.045) and N/L ratio (p = 0.047) were associated with the need for mechanical ventilation. Seven patients (32%) died during admission, including 86% (n = 6) of patients who received mechanical ventilation.

**Conclusions:**

In solid-organ transplant recipients with COVID-19, initial CRP and N/L ratio were associated with need for mechanical ventilation.

## Introduction

Coronavirus disease 2019 (COVID-19), caused by the Severe Acute Respiratory Syndrome Coronavirus 2 (SARS-CoV-2), continues to spread rapidly throughout the world. Much remains unknown about the effects of SARS-Cov-2 on organ function and the host response. The pathogenesis of COVID-19 induced organ dysfunction has been associated with evidence of a dysregulated immune system.^1^ Given that organ transplant recipients receive chronic immunosuppression, studying the course of disease and factors that associate with illness severity in this population may yield important observations. Literature describing COVID-19 in this population has grown since the pandemic began. This report will describe factors that are associated with critical illness in this population.

Reports from China and Spain first described a heart and renal transplant recipient, respectively, with COVID-19 at the beginning of the outbreak.^2,3^ Since then, several series describe outcomes in solid-organ transplant recipients with COVID-19.^4,5^ A larger study found solid-organ transplant recipients had worse outcomes with COVID-19 than non-transplanted patients, as has been shown for other infectious diseases.^6–8^

In this study, we describe clinical characteristics and outcomes of a cohort of solid-organ transplant patients admitted to a large health system during the first month of the COVID-19 outbreak in New York. We aim to determine which factors present at admission were associated with need for mechanical ventilation in the study population. We also seek to characterize alterations in lymphocyte count and kidney function in individual patients during COVID-19 infection as compared to their baseline values since these values are frequently abnormal in patients taking immunosuppressant medications or with renal transplantation.

## Methods

We performed a retrospective cohort study of all patients age 18 years and older with a history of solid-organ transplantation admitted to a Northwell Health hospital with a diagnosis of COVID-19 during the month of March 2020. Data collection closed after the last patient was discharged. Northwell is a 23-hospital network in the New York metropolitan area composed of community hospitals and tertiary care centers, with a total of over 4,000 beds.

We queried the electronic medical record for patients with a diagnosis of COVID-19 and history of solid-organ transplant within the study dates. Demographic, clinical, laboratory, radiologic, and management data were collected using a standardized data collection form by one investigator (FM). Clinical data collected included chief complaint, need for ICU admission, need for mechanical ventilation, length of stay, immunosuppressive medications, and COVID-19 specific treatments.

Laboratory values within 24 hours of admission recorded included white blood cell count, absolute lymphocyte count (ALC), absolute neutrophil count (ANC), neutrophil to lymphocyte ratio, creatinine, C-reactive protein (CRP), and ferritin. These labs were routinely measured in patients with COVID-19 at the study sites and were available for most patients. When available, baseline serum creatinine and ALC at least 30 days prior to admission and outside of an acute illness were also recorded. If baseline values were not available, the individual was excluded from that calculation. We defined acute kidney injury (AKI) via the Kidney Disease Improving Global Outcomes (KDIGO) classification system, comparing admission creatinine to a baseline level at least 30 days prior.^9^ For the cohort requiring mechanical ventilation, Acute Physiology and Chronic Health Evaluation II (APACHE-II) scores were calculated at time of ICU admission.^10^

We summarized continuous variables as means (standard deviation) or median (interquartile range, IQR), as appropriate. Categorical variables are reported as frequencies (percent). Student’s t-test was used to compare differences between groups’ baseline ALC and admission ALC, using each subject as a self-control.^11^ Odds ratios and confidence intervals for mechanical ventilation were calculated using logistic regression. A p-value of 0.05 was considered significant. Regression were performed with SAS: University-Edition (*SAS Institute, Cary, NC*).

## Results

### General Characteristics of Solid Organ Transplant Recipients Admitted for COVID-19

Over the 1-month study period, 22 patients met inclusion criteria (Table 1). The majority (77%, n = 17) were kidney transplant recipients. The remaining patients were recipients of liver (9%, n = 2), heart (9%, n = 2), and lung (5%, n = 1) transplants. Average time since transplantation was 93 ± 63 months. Presenting symptoms included fever (77%, n = 17), cough (50%, n = 11), dyspnea (32%, n = 7), and diarrhea (18%, n = 4). Thirty-six percent (n = 8) patients were admitted to the ICU and the remaining 64% (n = 14) were managed on the general inpatient floors. Indication for ICU admission in all patients was hypoxemic respiratory failure. Seven (32%) patients required mechanical ventilation. The median APACHE-II score of patients in our cohort requiring mechanical ventilation was 27 (IQR 21-34).

**Table 1.** Baseline Characteristics of the Study Cohort.

	Total cohort (n = 22)	Non-MV (n = 15)	MV (n = 7)
**Age (years, mean [median, STD])**	58.5 ± 14.0	56.5 ± 13.9	62.6 ± 14.3
**Sex (n, %)**			
Female	8 (36)	6 (40)	2 (29)
Male	14 (64)	9 (60)	5 (71)
**Organ Transplant Type (n, %)**			
Kidney	17 (77)	11 (73)	6 (86)
Liver	2 (9)	2 (13)	0
Lung	1 (5)	0	1 (14)
Heart	2 (9)	2 (13)	0
**Months Since Transplant (mean, STD)**	93 ± 63)	89 ± 55	102 ± 82
**Immunosuppressive Agents (n, %)**			
Tacrolimus	21 (95)	13 (87)	7 (100)
Mycophenolate Mofetil	20 (91)	13 (87)	7 (100)
Prednisone	15 (68)	9 (60)	6 (86)
Cyclosporine	1 (5)	1 (7)	0

Of the total cohort, 32% (n = 7) died and 68% (n = 15) survived to hospital discharge. Average length of stay (LOS) for survivors was 11.1 ± 6.7 days (Table 2). Six of 7 patients who died were kidney transplant recipients and one had received a lung transplant. Five of 7 mortalities were male (71%). All were prescribed tacrolimus and a mycophenolic acid derivative, and 6 of 7 were prescribed prednisone for immunosuppression. Additional information on patients requiring mechanical ventilation can be found in Table 3.

**Table 2.** Clinical Findings and Treatment Course of the Study Cohort.

	Total cohort (n = 22)	Non-MV (n = 15)	MV (n = 7)
**Presenting Signs and Symptoms (n, %)**			
Fever	17 (77)	12 80)	5 (71)
Cough	11 (50)	9 (60)	2 (29)
Dyspnea	7 (32)	4 (27)	3 (43)
Diarrhea	4 (18)	4 (27)	0
Hypoxemia	16 (73)	9 (60)	7 (100)
**APACHE-II (median, IQR)**			27 (21-34)
**ICU Admission (n, %)**	8 (36)	1 (7)	7 (100)
**COVID-19 Specific Treatments (n, %)**			
Hydroxychloroquine	17 (77)	11 (73)	6 (86)
Azithromycin	10 (45)	4 (27)	6 (86)
Corticosteroids	3 (14)	2 (13)	1 (14)
Tocilizumab	1 (5)	0	1 (14)
Anakinra	1 (5)	1 (7)	0
**Length of Stay (days, mean, SD)** ^**1**^	11.1 ± 6.7		
**Vital Status (n, %)**			
Alive	15 (68)	14 (93)	1 (14)
Deceased	7 (32)	1 (7)	6 (86)

^1^Calculated for patients discharged alive.

**Table 3.** Specific Treatment and Clinical Course Data for the 7 Patients Who Required Mechanical Ventilation.

Age	Sex	Organ transplanted	Months since transplant	Immunosuppression	LOS (days)	ICU LOS (days)	Outcome
40	M	Kidney	105	MMF, tacrolimus, prednisone	43	37	Died
80	M	Kidney	168	MMF, tacrolimus	20	14	Survived
57	M	Kidney	12	MMF, tacrolimus, prednisone	14	14	Died
56	M	Kidney	244	MMF, tacrolimus, prednisone	7	4	Died
56	F	Kidney	36	MMF, tacrolimus, prednisone	9	2	Died
77	M	Kidney	46	MMF, tacrolimus, prednisone	17	6	Died
72	F	Lung	106	MMF, tacrolimus, prednisone	54	44	Died

LOS, Length of Stay; ICU, Intensive Care Unit; MMF, Mycophenolate mofetil.

### Immunosuppression Regimens

All patients in our cohort were managed with immunosuppressive medications prior to hospital admission (Table 1). The most common immunosuppressants prior to admission were tacrolimus (95%, n = 21), mycophenolic acid derivatives (91%, n = 20) and prednisone (68%, n = 15). Mycophenolic acid derivatives were discontinued on all patients upon admission. Tacrolimus was discontinued in 10% (n = 2 of 21) and cyclosporine was discontinued in a liver-transplant patient for whom it was the sole immunosuppressant. All 14 patients on corticosteroids prior to admission were continued on corticosteroids at their home dose for the duration of admission. No acute episodes of rejection were treated during hospitalization for COVID-19.

### Laboratory Abnormalities

We compared ALC in solid organ transplant recipients during their COVID-19 infection to their baseline ALC (data available for 68%, n = 15). ALC was significantly lower during COVID-19 infection as compared to baseline (0.83 ± 0.47 vs 1.46 ± 0.73, p < 0.001).

**Figure 1.**
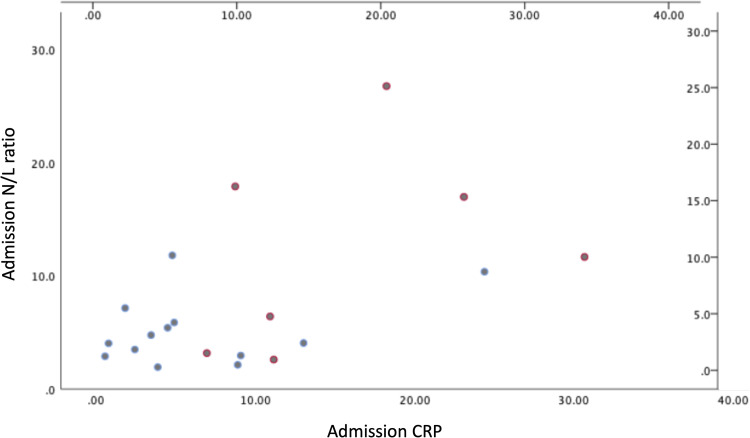
A plot of the admission CRP (mg/dL) level against the admission N/L ratio for patients who required and did not require mechanical ventilation.

We compared patients requiring mechanical ventilation to those not requiring mechanical ventilation, to determine if specific laboratory abnormalities present on admission were associated with eventual need for mechanical ventilation. For every 1-unit increase in the N/L, the odds of eventual mechanical ventilation increased by 24% (OR 1.24; 95% CI 1.01-1.52, p = 0.047). For every 1mg/dL increase in CRP at time of admission to the hospital, the odds of eventual mechanical ventilation increased by 18% (OR 1.18; 95% CI: 1.01-1.39, p = 0.045) (Figure 1). There was no association with admission WBC or ferritin and mechanical ventilation (Table 4).

**Table 4.** Laboratory Results of Patients on Admission.^a^

	Total Cohort (n = 22)	Non-MV (n = 15)	MV (n = 7)	OR (CI, p-value)	Reference Range
**Laboratory Values (mean, SD)**					
Leukocyte Counts					
White Blood Cell Count (n = 22)	5.9 ± 2.5	5.7 ± 2.1	5.4 ± 3.4	1.10 (0.77-1.58, 0.59)	3.8-10.5 K/μL
ANC (K/µL)	4.4 ± 2.3	4.0 ± 1.6	5.4 ± 3.2		1.8-7.4 K/μL
ALC (K/µL)	0.82 ± 0.4	0.91 ± 0.4	0.62 ± 0.4		1.0-3.3 K/μL
N:L	7.5 ± 6.4	5.3 ± 3.3	12.2 ± 8.9	1.24 (1.003-1.519, 0.047)	
CRP					<5.0 mg/L
Admission CRP (mg/dL)	9.6 ± 8.5 (n = 20)	6.33 ± 6.5 (n = 13)	15.7 ± 8.7	1.18 (1.004-1.387, 0.045)	
Ferritin					30-400 ng/mL
Admission ferritin (ng/mL)	1327 ± 1672 (n = 19)	1160 ± 1689 (n = 12)	1612 ± 1733	1.00 (1.00-1.00, 0.57)	
Creatinine					0.5-1.3 mg/dL
Admission Creatinine (mg/dL)	1.82 ± 1.07	1.72 ± 1.00	2.06 ± 1.26		
AKI on Admission (n, %)	7 (37%, n = 19)	4 (29%, n = 14)	3 (60%, n = 5)		

^a^ Odds ratios are for mechanical ventilation.

### Acute Kidney Injury

Rate of AKI in the total cohort of patients was 37% (n = 7, data available for 19 subjects). In patients requiring mechanical ventilation, the rate of AKI during their ICU stay was 60% (n = 3, data available for 5 of 7 patients).

### COVID-19 Specific Therapies

Hydroxychloroquine was administered to 77% (n = 17). Azithromycin was given to 41% (n = 9), 8 of whom also received hydroxychloroquine. Corticosteroids were given to 14% (n = 3). One patient (5%) received tocilizumab and one patient (5%) received anakinra.

## Discussion

We found typical features of COVID-19 presentation in our study cohort, including fever, cough, dyspnea, and lymphopenia. This is in agreement with findings of other studies of COVID-19 in solid organ transplant recipients.^4,6^ The neutrophil to lymphocyte ratio has previously been reported as a significant predictor of outcomes in COVID-19.^12^ We found that this value was also associated with the need for mechanical ventilation in solid-organ transplant recipients.

Lymphopenia has previously been associated with mortality in solid-organ transplant recipients and in general populations with COVID-19.^7,12–14^ It is unclear how the lymphopenia and T-cell dysfunction of immunosuppressed solid-organ transplant recipients affects the clinical course of COVID-19.^15,16^ We found reductions in ALC on admission with COVID-19 in patients with already impaired lymphocytes. Admission creatinine was higher in patients who required mechanical ventilation, this is similar to a recent large review of outcomes in COVID-19 in patients with solid organ transplants.^17^

CRP has been correlated to more severe illness in general adult studies with sepsis and COVID-19, specifically.^18,19^ We found CRP correlated with need for mechanical ventilation in our cohort. The values we report for CRP in our solid organ transplant recipient population are similar to levels reported in a general cohort of critically ill Chinese patients with COVID-19 = (average CRP 10.5 ± 1.27 mg/dL), as well as reported critically ill solid-organ transplant patients in New York.^6,20^

Given the small size of our cohort, we are unable to stratify based on type of organ transplantation or immunosuppressant regimen, though there may be differential effects of COVID-19 based on the transplanted organ. We report only one lung transplant recipient in our cohort and no pancreas or small intestine transplant recipients, though these are less common.

Future studies may determine if N/L and CRP have predictive value in the early identification of patients at risk for a more severe COVID-19 phenotype and subsequently most likely to benefit from COVID-19-specific interventions. This is especially valuable today given emerging evidence that convalescent plasma, remdesivir, and glucocorticoids may benefit patients with COVID-19.^21–23^ Identifying patients at risk of decline during admission could help direct future scarce therapies, better allocate resources, and inform patients and clinicians in shared decision making and prognosis. Much is still unknown regarding different immune responses to COVID-19 that may drive severity of infection. Patients with solid-organ transplants and associated T-cell suppression may have responses different from the general population that put them at different risk of developing severe disease. There is evidence that the CRP level can predict responsiveness to corticosteroids in a general population of hospitalized patients with COVID-19.^24^

Limitations of our study include its retrospective design and small sample size due to the recent emergences of COVID-19. The study’s descriptive nature precludes causal inference. Our method of identifying patients relied on electronic medical record capture of both COVID-19 and history of transplantation. As such, some transplant patients may not have been included in the study due to unknown errors in diagnostic coding. Despite these limitations, the association between elevations in N/L and CRP and need for mechanical ventilation reflect hypothesis generation that may warrant further exploration in solid organ transplant patients with COVID-19.

## Conclusions

Initial abnormalities in the CRP level and neutrophil to lymphocyte ratio are associated with subsequent mechanical ventilation in solid-organ transplant recipients.

## References

[1] Qin C Zhou L Hu Z. Dysregulation of immune response in patients with coronavirus 2019 (COVID-19) in Wuhan, China. Clin Infect Dis. 2020;71(15):762–768. doi:10.1093/cid/ciaa248 32161940 10.1093/cid/ciaa248 PMC7108125

[2] Li F Cai J Dong N. First cases of COVID-19 in heart transplantation from China. J Heart Lung Transpl. 2020;39(5):496–497. doi:10.1016/j.healun.2020.03.006 10.1016/j.healun.2020.03.006 PMC7156127 32362394

[3] Guillen E Pineiro GJ Revuelta I, et al. Case report of COVID-19 in a kidney transplant recipient: does immunosuppression alter the clinical presentation? Am J Transpl. 2020;20(7):1875–1878. doi:10.1111/ajt.15874 10.1111/ajt.15874 PMC7228209 32198834

[4] Nair V Jandovitz N Hirsch JS, et al. COVID-19 in kidney transplant recipients. Am J Transpl. 2020;20(7):1819–1825. doi:10.1111/ajt.15967 10.1111/ajt.15967 PMC7267603 32351040

[5] Yi SG Rogers AW Saharia A, et al. Early experience with COVID-19 and solid organ transplantation at a US high-volume transplant center. Transplantation. 2020;104(11):2208–2214. doi:10.1097/TP.0000000000003339 32496357 10.1097/TP.0000000000003339 PMC7302089

[6] Pereira MR Mohan S Cohen DJ, et al. COVID-19 in solid organ transplant recipients: initial report from the US epicenter [Published online May 10, 2020]. Am J Transpl. 2020. doi:10.1111/ajt.15941 10.1111/ajt.15941 PMC7264777 32330343

[7] Gotur DB Masud FN Ezeana CF, et al. Sepsis outcomes in solid organ transplant recipients. Transpl Infect Dis. 2020;22(1). doi:10.1111/tid.13214 10.1111/tid.13214 31755202

[8] Kalil AC Syed A Rupp ME, et al. Is bacteremic sepsis associated with higher mortality in transplant recipients than in nontransplant patients? A matched case-control propensity-adjusted study. Clin Infect Dis Off Publ Infect Dis Soc Am. 2015;60(2):216–222. doi:10.1093/cid/ciu789 10.1093/cid/ciu789 25301215

[9] Kellum JA Lameire N Aspelin P. Diagnosis, evaluation, and management of acute kidney injury: a KDIGO summary (Part 1). Crit Care. 2013;17(1):1–15. doi:10.1186/cc11454 10.1186/cc11454 PMC4057151 23394211

[10] Knaus WA Draper EA Wagner DP Zimmerman JE. APACHE II: a severity of disease classification system. Crit Care Med. 1985;13(10):818–829. 3928249

[11] Louis TA Lavori PW Bailar JC Polansky M. Crossover and self-controlled designs in clinical research. N Engl J Med. 1984;310(1):24–31. doi:10.1056/NEJM198401053100106 6689736 10.1056/NEJM198401053100106

[12] Nierenberg NE Poutsiaka DD Chow JK, et al. Pretransplant lymphopenia is a novel prognostic factor in CMV and non-CMV invasive infection after liver transplantation. Liver Transpl. 2014;20(12): 1497–1507. doi:10.1002/lt.23991 25205044 10.1002/lt.23991 PMC4451230

[13] Wan Q Zhang P Ye Q Zhou J. Acute respiratory distress syndrome in kidney transplant recipients. Intensive Care Med. 2014;41(2):373–374. doi:10.1007/s00134-014-3590-3 25465910 10.1007/s00134-014-3590-3 PMC7095300

[14] Tan L Wang Q Zhang D, et al. Lymphopenia predicts disease severity of COVID-19: a descriptive and predictive study. Signal Transduct Target Ther. 2020;5(1):1–3. doi:10.1038/s41392-020-0148-4 32296069 10.1038/s41392-020-0148-4 PMC7100419

[15] Zheng M Gao Y Wang G, et al. Functional exhaustion of antiviral lymphocytes in COVID-19 patients. Cell Mol Immunol. 2020;17(5):533-535. doi:10.1038/s41423-020-0402-2 10.1038/s41423-020-0402-2 PMC7091858 32203188

[16] Moon C. Fighting COVID-19 exhausts T cells [Published online April 6, 2020]. Nat Rev Immunol. 2020. doi:10.1038/s41577-020-0304-7 10.1038/s41577-020-0304-7 PMC7132550 32249845

[17] Molnar MZ Bhalla A Azhar A, et al. Outcomes of critically ill solid organ transplant patients with COVID-19 in the United States. Am J Transpl. 2020;20(11):3061–3071. doi:10.1111/ajt.16280 10.1111/ajt.16280 PMC7460925 32844546

[18] Koozi H Lengquist M Frigyesi A. C-reactive protein as a prognostic factor in intensive care admissions for sepsis: a Swedish Multicenter Study. J Crit Care. 2020;56:73–79. doi:10.1016/j.jcrc.2019.12.009 31855709 10.1016/j.jcrc.2019.12.009

[19] Huang C Wang Y Li X, et al. Clinical features of patients infected with 2019 novel coronavirus in Wuhan, China. The Lancet. 2020;395(10223):497–506. doi:10.1016/S0140-6736(20)30183-5 10.1016/S0140-6736(20)30183-5 PMC7159299 31986264

[20] Wang L. C-reactive protein levels in the early stage of COVID-19. Médecine Mal Infect. 2020;50(4):332–334. doi:10.1016/j.medmal.2020.03.007 10.1016/j.medmal.2020.03.007 PMC7146693 32243911

[21] Li L Zhang W Hu Y, et al. Effect of convalescent plasma therapy on time to clinical improvement in patients with severe and life-threatening COVID-19: a randomized clinical trial. JAMA. 2020;324(5):460–470. doi:10.1001/jama.2020.10044 32492084 10.1001/jama.2020.10044 PMC7270883

[22] Beigel JH Tomashek KM Dodd LE, et al. Remdesivir for the treatment of Covid-19—preliminary report. N Engl J Med. 2020;0(0): null. doi:10.1056/NEJMoa2007764 10.1056/NEJMc2022236 32649078

[23] Dexamethasone in Hospitalized Patients with Covid-19—Preliminary Report. N Engl J Med. 2020;0(0):null. doi:10.1056/NEJMoa2021436 10.1056/NEJMoa2021436 PMC7383595 32678530

[24] Keller J Kitsis E Arora S, et al. Effect of systemic glucocorticoids on mortality or mechanical ventilation in patients With COVID-19. J Hosp Med. 2020;15(8)489–493. doi:10.12788/jhm.3497. July 22 JHM 2020 A-493 POF, DOI 10.12788/jhm.3497 2020|. 32804611 10.12788/jhm.3497 PMC7518134

